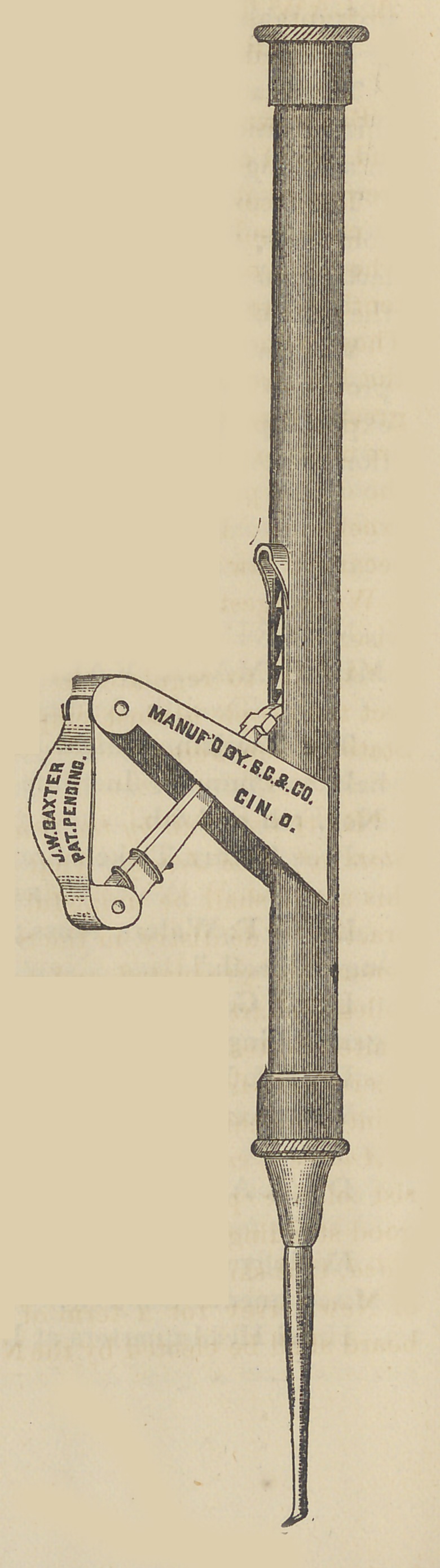# Baxter’s Automatic Mallet

**Published:** 1874-11

**Authors:** 


### BAXTER'S AUTOMATIC MALLET.

We have used for a short time Baxter's latest improved Automatic Mallet. It is certainly the most simple in construction and more efficient in operation than any one we have ever used. Its simplicity is apparent at first view; it is easily manipulated, being light and of convenient form, and a blow of any desirable force can be given without any change of the instrument or its position in the hand. Every one accustomed to the Automatic Mallet would certainly appreciate this instrument.

This mallet is the result of many years experiment and thought by Dr. Baxter upon this subject. He was the originator of the idea, and the first to make a practical automatic mallet for filling teeth; and all the forms and varieties of this instrument that have been produced during the last fifteen years are outgrowths from this first conception and production of Dr. Baxter, so far at least as we have been able to learn. If we are in error about this we shall be glad to be corrected, and will make the correction through the same channel as this is made. Now if any one has ought to say, "let him speak, or forever hold his peace"

The plugger is so well represented in the accompanying cut as to need no particular description. It is manufactured by our friends, Mess. Spencer, Crocker and Co., and, we presume, can be obtained at the dental depots.

## Figures and Tables

**Figure f1:**